# Analysis of Research Trends and Output Effectiveness in the Medical Field Based on the National Natural Science Foundation of China

**DOI:** 10.1002/hcs2.70075

**Published:** 2026-05-10

**Authors:** Hu Jin, Huang Hui, Bao Xinjie, Bai Hua

**Affiliations:** ^1^ Department of Scientific Research, Peking Union Medical College Hospital Chinese Academy of Medical Sciences & Peking Union Medical College Beijing China; ^2^ Comprehensive Office, Institute of Clinical Medicine Chinese Academy of Medical Sciences & Peking Union Medical College Beijing China

**Keywords:** compound annual growth rate, medical field, National Natural Science Foundation of China, optimal allocation of resources, output‐to‐project ratio

## Abstract

**Background:**

This study aims to analyze the trends in research focus and output effectiveness in the medical field supported by the National Natural Science Foundation of China (NSFC) over the past 15 years, providing a basis for the optimal allocation of scientific research resources.

**Methods:**

Based on the NSFC Big Data Knowledge Management Service Database, a retrospective approach was adopted to retrieve data on completed projects and output achievements from 2009 to 2023. Indicators, including compound annual growth rate (CAGR) and output‐to‐project ratio, were used to evaluate the growth of project completion and research productivity across 35 secondary medical categories.

**Results:**

Over the 15‐year period, a total of 439,637 NSFC projects were completed, with the Division of Medical Science ranking first in both scale (23%) and growth rate (CAGR 11%). The 35 secondary categories (H01–H35) under the Division of Medical Science showed differentiated development characteristics, namely “a few growing rapidly, most growing steadily, and a small number lagging behind”: six directions including biomedical engineering had a CAGR of ≥ 15%; oncology and traditional chinese medicine contributed large output volumes but showed low output‐to‐project ratios, whereas neuroscience, imaging medicine, and biomedical engineering achieved high efficiency. Key Programs achieved the highest output‐to‐project ratios, followed by the General Program and then the Young Scientists Fund.

**Conclusions:**

The completion and output of NSFC‐funded medical projects reflect national strategic orientations and disciplinary development laws, but imbalance remains between project scale and output efficiency across subfields. It is suggested to establish a dynamic evaluation mechanism, promote the “basic research‐application‐transformation” collaborative model, support small‐scale yet high‐efficiency fields, and conduct long‐term tracking of output achievements. These measures are expected to optimize the allocation of NSFC funding resources.

## Graphical abstract

The completion and output of NSFC‐funded medical projects reflect national strategic orientations and disciplinary development laws. It is suggested to establish a dynamic evaluation mechanism, promote the “basic research‐application‐transformation” collaborative model, support small‐scale yet high‐efficiency fields, and conduct long‐term tracking of output achievements. These measures are expected to optimize the allocation of NSFC funding resources.

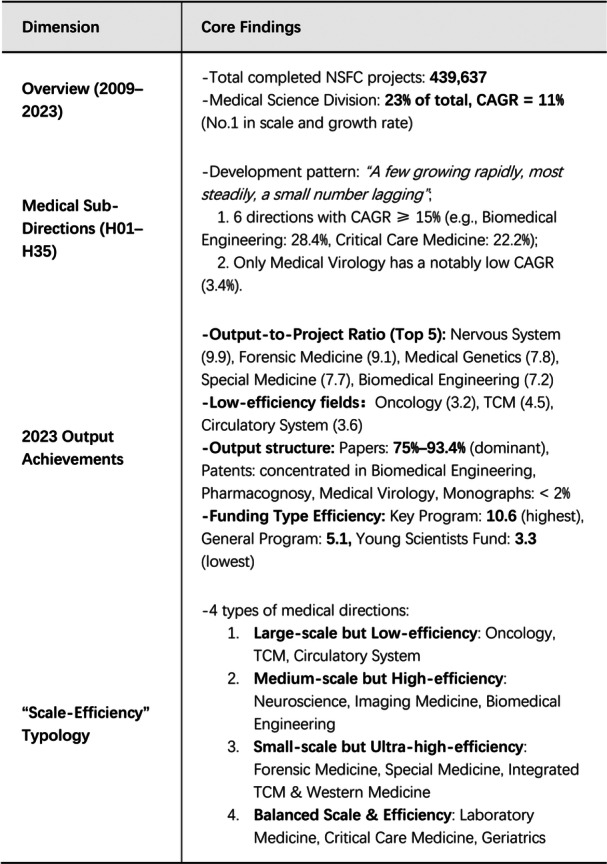

**Abbreviations**
CAGR: compound annual growth rate
NSFC: National Natural Science Foundation of China
TCM: traditional Chinese medicine

## 1 Background

Since its establishment in 1986, the National Natural Science Foundation of China (NSFC) has always been the core channel for supporting basic research and original innovation in China. It funds disciplinary fields including medicine, life sciences, engineering and materials sciences, information sciences, mathematics and physics sciences, and management sciences. It provides financial support and innovation platforms for researchers, helping China break through frontiers in basic research and cultivate high‐level talents [1]. In recent years, some domestic scholars in China have published several studies on NSFC funding in sub‐fields of medical fields, such as geriatrics [2], clinical translation of oncology [3], traditional Chinese medicine (TCM) [4, 5, 6], critical care medicine [7], ophthalmology [8], clinical pharmacology [9], and clinical/preventive medicine [10]. However, there is a lack of research on the overall landscape and project completion status of the entire medical field, making it difficult to fully present the development characteristics and output laws of the medical field. Therefore, based on the data from completed NSFC projects and output achievements in the medical field from 2009 to 2023, this study summarizes and analyzes the overall research trends and output effectiveness of the medical field. It not only helps to clearly grasp the dynamic development trends of the current medical field but also provides data support for optimizing NSFC's funding strategies for medical directions and improving the efficiency of scientific research achievement transformation.

## 2 Methods

### 2.1 Data Source

Based on the NSFC Big Data Knowledge Management Service Database [11], data on the number of completed projects and output achievements were retrieved retrospectively from 2009 to 2023 for all divisions and the 35 secondary categories (H01–H35) under the Division of Medical Science (each application code represents a medical research direction; e.g., H01 for respiratory system, H02 for circulatory system, H03 for digestive system, and so on). Specifically:

1. Data on completed projects include General Program, Young Scientists Fund Program, Key Program, Major Research Plan, Regional Science Fund Program, and Special Fund Program.
2. Data on output achievements include journal papers, conference papers, patents, awards, and monographs.

### 2.2 Analysis Methods

GraphPad 10.0 software (GraphPad Software, San Diego, USA) was used for statistical analysis. Indicators of Compound Annual Growth Rate (CAGR) and output‐to‐project ratio were applied to analyze the trends of NSFC project completion and output effectiveness. The following formula (1) was used to determine CAGR, where *n* is the number of years of growth [12]:

(1) CAGR=((finalvalue/initialvalue)^(1/n)−1)×100%

The following formula (2) was used to determine the output‐to‐project ratio:

(2) Output−to−projectratio=(numberofoutputachievements/numberofcompletedprojects)

## 3 Results

### 3.1 Analysis of NSFC Project Completion Status

#### 3.1.1 Distribution by Division

As shown in Table 1, over the 15 years from 2009 to 2023, the total number of completed projects maintained steady growth. A total of 439,637 projects were completed across all divisions, with the highest number reaching 41,820 in 2023. The overall CAGR was nearly 9%, reflecting NSFC's continuous support for scientific research.

**Table 1:** Distribution of completed NSFC projects by division.

Division	Number in 2023	Cumulative number (2009–2023)	CAGR (%) (2009–2023)
Medical sciences	10,133	99,896	11.0
Engineering and materials sciences	6996	72,833	9.9
Life sciences	6389	69,138	8.0
Mathematics and physics sciences	4174	47,403	7.7
Information sciences	4560	47,313	9.5
Earth sciences	4002	42,310	8.8
Chemical sciences	3663	41,543	7.9
Management sciences	1903	19,201	8.8
Total	41,820	439,637	9.2

Abbreviations: CAGR, compound annual growth rate; NSFC, National Natural Science Foundation of China.

In terms of scale, the Division of Medical Science ranked first, accounting for 23%, followed by the Division of Engineering and Materials Sciences (17%) and the Division of Life Sciences (16%). The Division of Management Sciences had the smallest scale, accounting for 4%. This distribution is highly consistent with national development strategies such as “Healthy China” and “Manufacturing Powerhouse”.

In terms of CAGR, the Division of Medical Science (11%) ranked first, followed by the Division of Engineering and Materials Sciences (9.9%) and the Division of Information Sciences (9.5%). These three divisions are all application‐oriented fields closely linked to practical needs (e.g., medical and health care, high‐end manufacturing, new materials, and digital economy). Their high growth rates reflect the dual driving forces of national policy support/guidance and market demand. The Division of Mathematics and Physics Sciences (7.7%), Division of Life Sciences (8.0%), and Division of Chemical Sciences (7.9%) focus on basic research, which typically involves long research and development cycles and slow achievement transformation—consistent with industry rules and requiring long‐term stable support. The Division of Management Sciences showed a “small but stable” pattern: it had the smallest scale but a growth rate close to the overall average (8.8%).

#### 3.1.2 Distribution of Secondary Categories Under the Division of Medical Science

As shown in Table 2, the CAGR of various medical directions under the Division of Medical Science from 2009 to 2023 ranged from 3.4% to 28.4%, presenting a pattern of “a few directions growing at a high speed, most growing steadily, and a small number lagging behind”. The specific characteristics are as follows:

1. Outstanding high‐growth directions
  There were six directions with a CAGR of ≥ 15%, which are the most dynamically growing segments in medical research. The top five directions were biomedical engineering (28.4%), critical care medicine (22.2%), geriatrics (21.1%), TCM pharmacy (18.4%), and laboratory medicine (18.3%). The high growth of these fields may be attributed to national policy support, technological progress (e.g., AI‐assisted diagnosis and treatment, high‐end medical devices, precision diagnosis and treatment, critical care, and inheritance of TCM), public health emergencies, and the intensification of population aging.
2. Concentrated medium‐growth directions
  There were 18 directions with a CAGR between 10.3% and 14.8%, accounting for more than half of all directions. Examples include rehabilitation medicine (14.8%), digestive system (14.2%), reproductive system/perinatology/neonatology (13.4%), endocrine system (12.9%), and oncology (12.2%). These medical directions mainly focus on common and frequently occurring clinical diseases, closely related to daily clinical diagnosis and treatment. With mature research directions, they form the “basic core” of medical research and are less affected by short‐term policies or technological shocks, thus maintaining a medium growth rate.
3. Few low‐growth directions
  There were 11 directions with a CAGR of < 10%, most of which were concentrated in the range of 6%–9%. Only medical virology (3.4%) had a significantly lower growth rate. Other representative directions with low growth included trauma/burn/plastic surgery, preventive medicine, medical immunology, and neuroscience. Most of these directions belong to basic research or traditional technology fields; their slow growth is mainly affected by factors such as long research cycles, technological bottlenecks, and relatively stable demand.

**Table 2:** The CAGR of various medical directions under the division of medical science from 2009 to 2023.

Code	Directions	CAGR (%)
H28	Biomedical engineering	28.4
H16	Critical care medicine	22.2
H19	Geriatrics	21.1
H32	TCM pharmacy	18.4
H26	Laboratory medicine	18.3
H10	Mental health and psychology	15.5
H20	Rehabilitation medicine	14.8
H3	Digestive system	14.2
H4	Reproductive system/perinatology/newborns	13.4
H14	Otorhinolaryngology—head and neck surgery	13.1
H7	Endocrine system/metabolic nutrition	12.9
H12	Dermatology	12.4
H18	Oncology	12.2
H5	Urinary system	11.8
H13	Ophthalmology	11.6
H27	Imaging medicine/nuclear medicine	11.4
H23	Medical genetics	11.3
H24	Special medicine	11.3
H31	TCM	11.3
H1	Respiratory system	10.9
H6	Locomotor system	10.7
H2	Circulatory system	10.6
H15	Oral and craniomaxillofacial science	10.6
H8	Hematological system	10.3
H34	Pharmacognosy	9.8
H25	Forensic medicine	9.7
H22	Medical pathogenic organisms and infections	8.4
H35	Pharmacology	8.2
H33	Integrated traditional Chinese and Western medicine	8.0
H29	Radiation medicine	7.8
H17	Trauma/burn/plastic surgery	7.3
H30	Preventive medicine	7.0
H11	Medical immunology	6.4
H9	Nervous system	6.3
H21	Medical virology and viral infections	3.4

Abbreviations: CAGR, compound annual growth rate; TCM, traditional Chinese medicine.

### 3.2 Analysis of Output Effectiveness of Completed NSFC Projects in the Division of Medical Science

#### 3.2.1 Analysis of the Number of Output Achievements

Taking the latest data of 2023 as an example (Table 3), the 35 medical directions were divided into four tiers based on the number of output achievements: tier 1 (≥ 2000 items), tier 2 (1000–1999 items), tier 3 (500–999 items), and tier 4 (< 500 items). The total output achievements of the medical field were highly concentrated in the seven directions of Tier 1, among which oncology ranked first with 6172 achievements—far exceeding other directions.

**Table 3:** Distribution and characteristics of output achievements of various medical directions in the division of medical science (2023).

Tiers	Range	Representative directions (*n*)	Characteristics
Tier 1	≥ 2000 items	Oncology (6172), TCM (3083), nervous system (2556), imaging medicine (2316), circulatory system (2173), TCM pharmacy (2161), preventive medicine (2160)	7 directions in total, with 20,621 cumulative achievements accounting for approximately 50% of total achievements—serving as the core contributors to medical achievements.
Tier 2	1000–1999 items	Locomotor system (1366), pharmacognosy (1339), digestive system (1333), integrated TCM and western medicine (1270), reproductive system (1177), oral and craniomaxillofacial science (1166), etc.	8 directions in total, with 9722 cumulative achievements accounting for 24% of total achievements—acting as the backbone supporting force.
Tier 3	500–999 items	Medical immunology (993), ophthalmology (956), hematological system (947), biomedical engineering (923), urinary system (882), respiratory system (881), etc.	10 directions in total, with 7773 cumulative achievements accounting for 19% of total achievements—with limited output scale.
Tier 4	< 500 items	Otorhinolaryngology‐head and neck surgery (494), trauma/burn/plastic surgery (421), geriatrics (378), medical virology (321), radiation medicine (132), forensic medicine (182), special medicine (77), medical genetics (39)	10 directions in total, with 2889 cumulative achievements accounting for 7% of total achievements—mostly small‐scale directions.

*Note: n*, number of items.

Abbreviation: TCM, traditional Chinese medicine.

Across all secondary medical categories (Table 4), scholarly papers constitute the dominant form of output, consistently exceeding 75% and often surpassing 90%. In contrast, awards, patents, and monographs represent comparatively small proportions and vary more widely between fields. Categories such as forensic medicine, integrated traditional Chinese and western medicine, and medical genetics exhibit the highest concentration of paper‐based output. Conversely, fields with stronger applied or technological orientations—such as biomedical engineering, pharmacognosy, medical virology, and laboratory medicine—show relatively higher proportions of patent production. Monographs remain the least common output type across categories, generally accounting for less than 2%. Overall, the distribution suggests that research activity is heavily publication‐oriented, with specialized and innovation‐driven disciplines generating proportionally more patents than clinically or traditionally focused fields.

**Table 4:** Types of output achievements in 2023 (proportion of papers/awards/patents/monographs).

Code	Directions	Papers (%)	Awards (%)	Patents (%)	Monographs (%)
H25	Forensic medicine	93.4	1.6	3.3	1.6
H33	Integrated traditional Chinese and Western medicine	93.1	0.0	5.7	1.2
H05	Urinary system	92.5	2.8	4.6	0.0
H23	Medical genetics	92.3	5.1	2.6	0.0
H01	Respiratory system	91.6	3.7	3.7	0.9
H30	Preventive medicine	91.3	4.1	3.5	1.2
H24	Special medicine	90.9	0.0	7.8	1.3
H09	Nervous system	90.6	4.1	4.4	0.9
H03	Digestive system	90.5	4.1	4.7	0.7
H08	Hematological system	90.5	3.6	5.6	0.3
H10	Mental health and psychology	90.3	2.9	5.4	1.4
H07	Endocrine system/metabolic nutrition	89.9	4.4	5.2	0.5
H13	Ophthalmology	89.7	3.8	5.5	0.9
H16	Critical care medicine	89.4	3.5	5.9	1.2
H02	Circulatory system	88.9	5.0	5.2	0.9
H14	Otorhinolaryngology—head and neck surgery	88.7	3.8	6.5	1.0
H31	TCM	88.4	5.4	4.6	1.7
H18	Oncology	88.2	4.5	6.5	0.7
H12	Dermatology	88.0	4.3	7.0	0.8
H22	Medical pathogenic organisms and infections	87.7	3.4	6.7	2.2
H20	Rehabilitation medicine	86.3	8.0	4.3	1.4
H04	Reproductive system/perinatology/newborns	86.3	5.7	7.4	0.6
H27	Imaging medicine/nuclear medicine	86.1	4.4	8.4	1.0
H15	Oral and craniomaxillofacial science	85.9	4.9	8.0	1.2
H11	Medical immunology	85.7	5.1	8.4	0.8
H17	Trauma/burn/plastic surgery	85.5	3.1	10.9	0.5
H06	Locomotor system	83.5	6.4	9.4	0.7
H21	Medical virology and viral infections	83.2	2.2	14.6	0.0
H32	TCM pharmacy	82.9	5.5	10.4	1.2
H35	Pharmacology	82.3	4.3	12.5	0.8
H29	Radiation medicine	81.8	6.8	9.8	1.5
H26	Laboratory medicine	81.8	3.6	12.8	1.7
H19	Geriatrics	81.7	4.2	13.5	0.5
H34	Pharmacognosy	80.3	3.2	16.1	0.4
H28	Biomedical engineering	76.1	5.5	17.7	0.8

Abbreviation: TCM, traditional Chinese medicine.

#### 3.2.2 Analysis of Output‐to‐Project Ratio

As shown in Table 5, the top five medical directions in terms of output‐to‐project ratio were: nervous system (9.9), forensic medicine (9.1), medical genetics (7.8), special medicine (7.7), and biomedical engineering (7.2). Directions with low output‐to‐project ratio included: oncology (3.2), pharmacology (3.3), urinary system (3.5), circulatory system, and medical pathogenic organisms and infections (3.6).

**Table 5:** Output‐to‐project status of completed NSFC projects in the division of medical science (2023).

Code	Directions	*n*	Output‐to‐project ratio
H18	Oncology	6172	3.2
H31	TCM	3083	4.5
H09	Nervous system	2556	9.9
H27	Imaging medicine/nuclear medicine	2316	6.4
H02	Circulatory system	2173	3.6
H32	TCM pharmacy	2161	4.4
H30	Preventive medicine	2160	5.4
H06	Locomotor system	1366	4.7
H34	Pharmacognosy	1339	4.1
H03	Digestive system	1333	4.0
H33	Integrated traditional Chinese and Western medicine	1270	6.9
H04	Reproductive system/perinatology/newborns	1177	3.5
H15	Oral and craniomaxillofacial science	1166	5.0
H35	Pharmacology	1068	3.3
H07	Endocrine system/metabolic nutrition	1003	3.7
H11	Medical immunology	993	3.8
H13	Ophthalmology	956	4.6
H08	Hematological system	947	3.6
H28	Biomedical engineering	923	7.2
H05	Urinary system	882	3.5
H01	Respiratory system	881	3.7
H10	Mental health and psychology	579	4.4
H16	Critical care medicine	575	4.1
H26	Laboratory medicine	522	4.2
H12	Dermatology	515	3.9
H14	Otorhinolaryngology—head and neck surgery	494	4.3
H22	Medical pathogenic organisms and infections	494	3.6
H17	Trauma/burn/plastic surgery	421	4.7
H19	Geriatrics	378	4.3
H20	Rehabilitation medicine	351	4.9
H21	Medical virology and viral infections	321	3.7
H25	Forensic medicine	182	9.1
H29	Radiation medicine	132	3.9
H24	Special medicine	77	7.7
H23	Medical genetics	39	7.8

*Note: n*, number of output achievements.

Abbreviations: NSFC, National Natural Science Foundation of China; TCM, traditional Chinese medicine.

We noted that the Nervous System shows a relatively low CAGR (Table 2), while the highest output‐to‐project ratio (Table 5). The main reason is that this field maintains stable funding intensity and benefits from strong accumulated research capacity, mature platforms, and well‐established methodologies, which enable significant total output despite low growth in project numbers.

Combining the above results of the number of output achievements (defined as “scale”) and output‐to‐project ratio (defined as “efficiency”), the medical directions were classified into four types to evaluate the effectiveness of resource input and achievement returns (Table 6).

**Table 6:** Effectiveness evaluation of completed NSFC Projects in the division of medical science (2023).

Type	Representative directions	Characteristics (*n*, %)^a^	Effectiveness evaluation
Large‐scale but low‐efficiency type	Oncology, TCM, TCM pharmacy, circulatory system	Ranked among the top in total achievements (6172/3083/2161/2173 items) but with low efficiency (3.2/4.5/4.4/3.6), relying on “large input for large output”	Make significant contributions to total output, but have low transformation efficiency per unit of resource—with room for optimization
Medium‐scale but high‐efficiency type	Nervous system, imaging medicine, biomedical engineering	Medium total achievements (2556/2316/923 items) and high efficiency (9.9/6.4/7.2), realizing “medium input for high output”	Precise resource input and optimal efficiency
Small‐scale but ultra‐high‐efficiency type	Integrated TCM and western medicine, forensic medicine, special medicine	Below‐medium total achievements (1270/182/77 items) but excellent efficiency (6.9/9.1/7.7), realizing “medium input for high returns”	Deep cultivation in small and sophisticated sub‐fields with extremely high resource utilization efficiency—can serve as efficiency benchmarks
Balanced scale and efficiency type	Laboratory medicine, critical care medicine, geriatrics, etc.	Balanced matching between total achievements (522/575/378 items) and efficiency (4.2/4.1/4.3)	Balanced efficiency and total output, with stable matching between resource input and output

Abbreviations: NSFC, National Natural Science Foundation of China; TCM, traditional Chinese medicine.

^a^ Characteristics are presented as the number of items (*n*) or ratios (%).

#### 3.2.3 Analysis of the Funding Types

As of Table 7, key programs exhibit the highest output‐to‐project ratios (10.6 overall), performing roughly double the general program (5.1) and more than three times the young scientists fund (3.3). This pattern holds across most secondary categories, reaffirming the key program's role in supporting larger‐scale, higher‐impact research. Across most secondary categories, the General Program maintains a stable mid‐range ratio (typically around 4–6), while the Young Scientists Fund consistently reports the lowest ratios (generally 2–4), indicating more modest outputs associated with early‐career funding.

**Table 7:** Output‐to‐project ratios comparison for different funding types in the division of medical science (2023).

Code	Directions	General program	Young scientists fund program	Key program
	Overall	5.1	3.3	10.6
H01	Respiratory system	5.0	2.8	12.0
H02	Circulatory system	4.7	2.7	7.7
H03	Digestive system	5.2	3.4	12.0
H04	Reproductive system/perinatology/newborns	4.6	2.8	7.2
H05	Urinary system	4.6	2.8	7.0
H06	Locomotor system	5.4	4.3	9.8
H07	Endocrine system/metabolic nutrition	4.9	3.0	11.5
H08	Hematological system	5.1	2.5	6.9
H09	Nervous system	5.2	2.9	9.4
H10	Mental health and psychology	5.4	3.9	36.0
H11	Medical immunology	4.7	2.9	11.0
H12	Dermatology	5.5	3.2	5.7
H13	Ophthalmology	6.2	3.5	12.0
H14	Otorhinolaryngology—head and neck surgery	5.6	3.7	5.7
H15	Oral and craniomaxillofacial science	7.0	4.0	28.0
H16	Critical care medicine	5.3	3.1	11.0
H17	Trauma/burn/plastic surgery	6.0	4.0	2.0
H18	Oncology	4.2	2.7	11.6
H19	Geriatrics	4.8	2.8	3.0
H20	Rehabilitation medicine	6.0	4.3	2.0
H21	Medical virology and viral infections	4.0	3.0	12.0
H22	Medical pathogenic organisms and infections	4.8	2.5	6.0
H23	Medical genetics	7.5	11.0	1.0
H24	Special medicine	4.1	3.7	2.5
H25	Forensic medicine	6.1	5.5	22.0
H26	Laboratory medicine	5.4	3.3	8.0
H27	Imaging medicine/nuclear medicine	8.2	4.9	16.3
H28	Biomedical engineering	7.6	6.8	33.0
H29	Radiation medicine	4.7	2.9	12.0
H30	Preventive medicine	6.5	4.8	15.0
H31	TCM	5.5	3.8	8.7
H32	TCM pharmacy	4.7	4.0	12.3
H33	Integrated traditional Chinese and Western medicine	4.7	3.0	8.0
H34	Pharmacognosy	4.7	3.8	28.0
H35	Pharmacology	4.3	2.6	7.1

Abbreviation: TCM, traditional Chinese medicine.

Overall, this distribution highlights the differentiated roles of the three funding types in advancing scientific research, with the Key Program driving high‐impact production, the General Program supporting broad and steady research activity, and the Young Scientists Fund nurturing emerging investigators.

## 4 Discussion

Data on completed NSFC projects from 2009 to 2023 show an overall trend of steady scale expansion. Differences in the scale and growth rate of completed projects across divisions reflect the deep integration of national strategic orientations for scientific research resource allocation and disciplinary development laws. In terms of NSFC funding, it is feasible to continuously adjust the proportion of resource allocation in line with major national development strategies, focus on the development of “chokepoint” technologies, and promote interdisciplinary and original research.

Regarding the output effectiveness of the Division of Medical Science, the low efficiency in fields such as oncology and TCM is not simply due to excessive input, but rather the lack of precise screening of research directions (e.g., avoiding homogeneous research). The top five research areas in oncology—such as tumor recurrence and metastasis, tumorigenesis, and tumor chemotherapy—together account for over 30% and scattered project approval [13]. The NSFC Guidelines for 2024 and 2025 also launched a pilot reform for general program applications in H18 Oncology and its subordinate codes, requiring applicants to highlight the innovation of the proposed project and previous innovative findings, showing that the NSFC agency has already recognized and addressed the risks of homogeneity—consistent with the results of this study [14, 15].

Directions relying on technological iteration, such as biomedical engineering and imaging medicine, have benefited from the full‐chain collaboration of “basic research‐technological development‐clinical transformation”, resulting in faster technological development and clinical transformation, and significantly higher output efficiency than other medical research directions. Small‐scale directions such as forensic medicine and special medicine have obvious advantages of being “small but sophisticated” and outstanding output efficiency.

Therefore, the following suggestions are put forward for optimizing the allocation of NSFC funding resources in the medical field:

1. For “large‐scale but low‐efficiency” research directions (e.g., oncology, TCM, TCM pharmacy, circulatory system): Establish a dynamic evaluation mechanism for research directions. Use big data to screen out duplicate research directions, guide resources to concentrate on sub‐frontiers and urgent clinical needs (e.g., tumor microenvironment, new targets for immunotherapy, advanced tumor treatment, and early screening), shorten the cycle of achievement transformation, and improve the output of achievements per project.
2. Promote the experience of “high‐efficiency” research directions: In fields such as biomedical engineering, imaging medicine, and neuroscience, summarize the “basic research‐application‐transformation” collaborative model, and establish a “Special Fund for Achievement Transformation” to accelerate technological implementation.
3. Support “small‐scale but high‐efficiency” research directions: Appropriately increase resource input for integrated TCM and Western Medicine, forensic medicine, and special medicine to expand the total amount of “high‐efficiency achievements”.
4. Establish a “long‐term tracking mechanism for output effectiveness”: Conduct long‐term tracking (for 5–10 years) of completed projects in various medical sub‐directions, evaluate the sustained impact of achievements (e.g., the contribution of oncology research to the revision of clinical guidelines), and provide a basis for subsequent resource allocation.

## 5 Conclusions

This study systematically analyzes the trends of project completion and output effectiveness in the medical field, and clarifies the development characteristics and resource allocation status of this field. The overall development trend of the medical field is highly consistent with national strategies such as “Healthy China” and social needs, including population aging and public health emergencies. However, some fields (e.g., oncology), although leading in achievement scale, have problems with low transformation efficiency per unit of resource input, and there is still room for optimizing funding strategies. The results of this study can provide practical references for NSFC to optimize funding strategies in the medical field, help improve the efficiency of scientific research resource utilization and the quality of achievement transformation, and promote medical research to better serve national health strategies and clinical needs.

## Author Contributions

**Jin Hu:** conceptualization, methodology, data curation, formal analysis, and writing – original draft. **Hui Huang:** writing – review and editing, project administration, and supervision. **Xinjie Bao:** writing – review and editing. **Hua Bai:** writing – review and editing, supervision, project administration, and validation.

## Funding

The authors have nothing to report.

## Ethics Statement

The authors have nothing to report.

## Consent

The authors have nothing to report.

## Conflicts of Interest

The authors declare no conflicts of interest.

## Data Availability

The data that support the findings of this study are openly available in National Natural Science Foundation of China at https://kd.nsfc.cn/supportStatistic.
